# Supplementary material for: Highly Sensitive Sub-ppm CH3COOH Detection by Improved Assembly of Sn3O4-RGO Nanocomposite
Source: Molecules. 2022 Dec 8;27(24):8707. doi: 10.3390/molecules27248707 (PMC9783866; doi:10.3390/molecules27248707)
Supplement: Supplementary file 1 [file molecules-27-08707-s001.zip › molecules-2023178-supplementary.pdf]

Supplementary Data

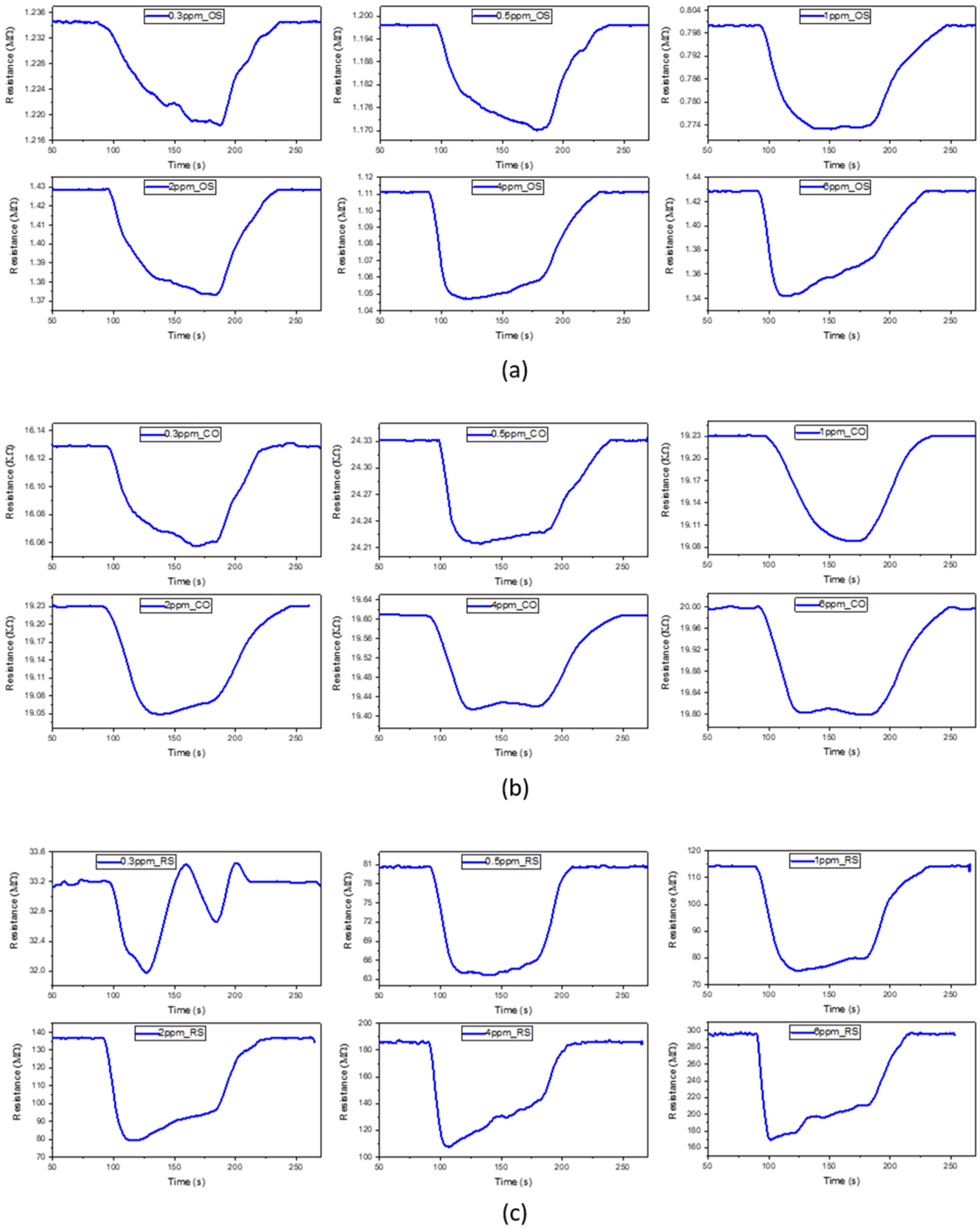

Figure S1. Resistance changes measured in the 0.3–6 ppm range of CH_3COOH exposure under a) OS nanocomposite, (b) CO nanocomposite and (c) RS nanocomposite sensor.
